# Reversing the Mismatch With Forefoot Striking to Reduce Running Injuries

**DOI:** 10.3389/fspor.2022.794005

**Published:** 2022-05-19

**Authors:** Irene S. Davis, Tony Lin-Wei Chen, Scott C. Wearing

**Affiliations:** ^1^Spaulding National Running Center, Department of Physical Medicine and Rehabilitation, Harvard Medical School, Cambridge, MA, United States; ^2^Department of Biomedical Engineering, Faculty of Engineering, The Hong Kong Polytechnic University, Hong Kong, Hong Kong SAR, China; ^3^Faculty of Sport and Health Sciences, Technical University of Munich, Munich, Germany; ^4^Faculty of Health, School of Clinical Sciences, Queensland University of Technology, Brisbane, QLD, Australia

**Keywords:** forefoot striking, mismatch theory of evolution, running injuries, running mechanics, foot structure and function

## Abstract

Recent studies have suggested that 95% of modern runners land with a rearfoot strike (RFS) pattern. However, we hypothesize that running with an RFS pattern is indicative of an evolutionary mismatch that can lead to musculoskeletal injury. This perspective is predicated on the notion that our ancestors evolved to run barefoot and primarily with a forefoot strike (FFS) pattern. We contend that structures of the foot and ankle are optimized for forefoot striking which likely led to this pattern in our barefoot state. We propose that the evolutionary mismatch today has been driven by modern footwear that has altered our footstrike pattern. In this paper, we review the differences in foot and ankle function during both a RFS and FFS running pattern. This is followed by a discussion of the interaction of footstrike and footwear on running mechanics. We present evidence supporting the benefits of forefoot striking with respect to common running injuries such as anterior compartment syndrome and patellofemoral pain syndrome. We review the importance of a gradual shift to FFS running to reduce transition-related injuries. In sum, we will make an evidence-based argument for the use of minimal footwear with a FFS pattern to optimize foot strength and function, minimize ground reaction force impacts and reduce injury risk.

## Introduction

**Figure 1:**
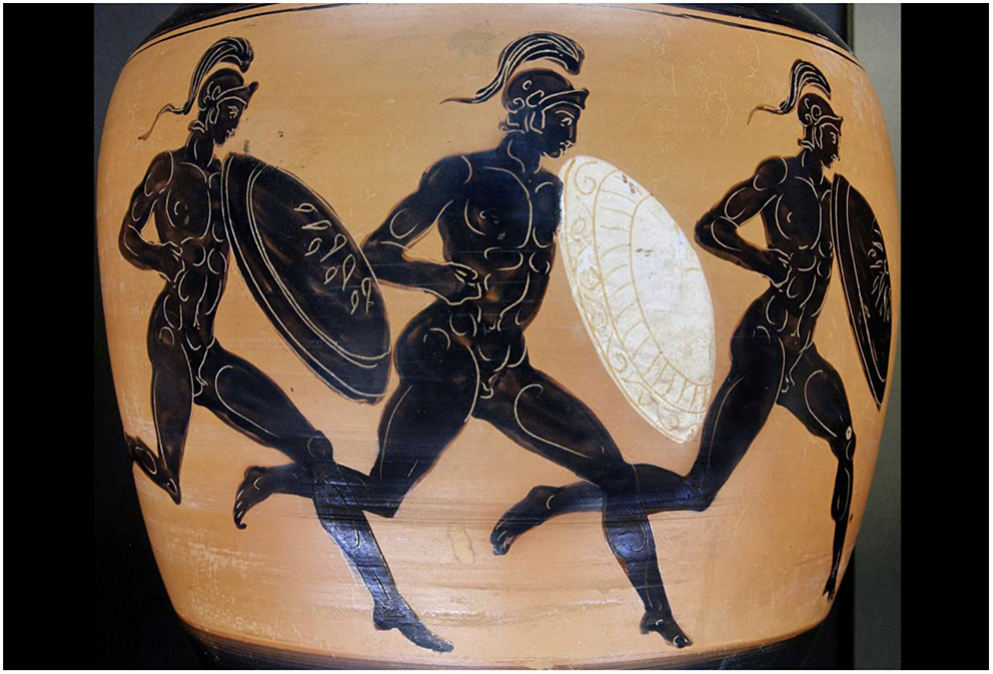
Depiction of runners during the Panatheniaic games 323-322 BC. Note the forefoot strike pattern of the runners. https://en.wikipedia.org/wiki/Hoplitodromos.

Early images of humans running depict them landing on their forefoot (Figure 1). However, there have been lively debates among scientists (Davis et al., 2017; Hamill and Gruber, 2017) and among the lay public^1^^,^^2^ regarding footstrike patterns in runners. These debates have focused on whether a forefoot strike (FFS) is more natural than a rearfoot strike (RFS). and whether it reduces the risk for injury. Unfortunately, there are no long-term observational studies to date to settle these debates. The purpose of this paper is to present the perspective that we evolved for forefoot strike running. As it is our most natural form, we propose that this will lead to reduced musculoskeletal injuries. We posit that our foot structure is optimized for a FFS pattern. We postulate that many of the injuries runners sustain are due to an evolutionary mismatch between the way we were adapted to run and the way we run today. Based upon the evidence, we believe that this mismatch is driven, in part, by the footwear we run in. However, it is important to understand that RFS runners habituated to modern cushioned and supportive shoes must make transitions in footwear and foot strike pattern gradually. A rapid change can lead to an overload of the musculoskeletal structures of the lower extremity resulting in a greater risk of injury. Starting our children in minimal footwear that promotes a FFS pattern in running avoids transition injuries and may reduce their overall risk for lower extremity musculoskeletal injuries.

## Mismatch Theory of Evolution

The mismatch theory of evolution, proposed by evolutionary biologists, states that our environment is changing faster than our bodies can adapt (Stearns, 1989; Lieberman, 2012). This creates a mismatch between the way our bodies have evolved and how they are being used today. While mismatches can be beneficial at times, they can also create health problems. For example, our overall activity level has been significantly reduced from that of our ancestors (Lieberman, 2012). This has contributed to the increased obesity we have as well as other non-communicable diseases such as diabetes and cardiovascular disease. More sedentary lifestyle is a mismatch to our biological systems. This mismatch can be extended to musculoskeletal injuries as well. For example, we evolved with strong lumbopelvic core musculature to support us during upright physical activities such as walking, running, climbing, etc. However, in our modern world, we spend 6–8 h sitting (Ussery et al., 2018), reducing the demand on our lumbopelvic core muscles and leading to their weakening. Weak lumbo-pelvic core muscles can lead to instability and faulty lower extremity mechanics. Chronic sitting is a mismatch for our lumbopelvic core muscles. Relevant to this paper, we propose that modern running footwear, as well as rearfoot strike running patterns have altered our foot function. This has led to mismatches to the way that our bodies were adapted to run, thereby increasing our risk for injury.

## We are Optimized to Run With a Forefoot Strike Pattern

### Tissue Characteristics

The human foot contains specialized fibroelastic fat pads beneath the heel and forefoot. The heel fat pad is thought to provide three main mechanical functions during ambulation (Aerts et al., 1996). The first is *shock reduction*, During the initial contact phase of walking, the fat pad undergoes considerable vertical deformation, approximately 10.3 ± 1.9 mm (Wearing et al., 2009, 2014) but the energy required to compress the heel pad (1.5 J) is relatively low (Wearing et al., 2009) compared to the impact energy of the foot and only about 1% of the total energy exchanged during walking (in a 70 kg adult walking at 4.5 km/h) (Cavagna et al., 2000). Hence, the initial loading rate of the heel pad during in running is extremely high [~250 kN.s^−1^] and the heel pad offers minimal resistance to deformation (Lieberman et al., 2015). This suggests it has only a minor shock reduction capacity during walking, let alone running. The second function of the heel pad is *protection against excessive plantar pressure*. Deformation of the heel pad also serves to increase the contact area and lower the peak pressure at the calcaneus, and thereby protecting it from injury (De Clercq et al., 1994). Estimated limits of pain tolerance for impacts of the heel pad equates to deformation of approximately 10.7 mm. The limit of pain tolerance for impacts involving the heel pad occurs at energy levels higher than 2.12 J (Cavanagh et al., 1984), which corresponds to a predicted heel pad deformation of 10.7 mm, which is marginally greater than that which typically occurs with walking (10.3 ± 1.9 mm) (Wearing et al., 2009, 2014). Hence, even at relatively slow walking speeds, deformation of the heel fat pad is close to the limits of pain tolerance. Thus, at higher gait speeds barefoot runners likely assume a more anterior footstrike pattern as a pain avoidance strategy. Indeed, Lieberman et al. have shown that the more time runners spend barefoot, the greater their tendency to run with a FFS pattern (Lieberman et al., 2015). The third function of the heel pad is *energy dissipation*. However, the energy dissipated by the heel pad is relatively low compared to other soft tissues of the foot and ankle (Wearing et al., 2014). This makes the heel pad a less than ideal structure for dissipating the impacts associated with running. Fibroelastic fat pads of the forefoot, on the other hand, have been shown to have a higher material stiffness and greater energy dissipation than those of the heel pad (Ledoux and Blevins, 2007; Pai and Ledoux, 2010; Chao et al., 2011). For instance, Pai and Ledoux (Pai and Ledoux, 2010) demonstrated that cadaveric fat pads of the forefoot were 20–35% stiffer and dissipated 10–20% more energy than those of the heel when tested under physiologically relevant cyclic loading conditions. This makes the forefoot pad more resistant to deformation but also more suited for attenuating loads of landing during running. This innate difference between the rearfoot and forefoot pads suggests that we are optimized to run with a FFS pattern.

The plantar fascia also plays an important role in the energetics of the foot during running. With deflection of the foot arch, the plantar fascia and associated deep ligaments of the foot are strained and subsequently return around 6% to 17% of the total mechanical work of running (Ker et al., 1987; Stearne et al., 2016). There is emerging evidence that a FFS pattern may induce greater deflection of the arch than RFS (Ker et al., 1987; Chao et al., 2011; Stearne et al., 2016), thus further increasing the peak strain within these structures. As such, a FFS pattern has greater potential to store and return elastic strain energy via the passive components of the arch than a RFS pattern, although this has yet to be determined. FFS runners have also been shown to have a greater volume and activation of the intrinsic foot muscles. This activation assists in the function of the plantar fascia, when compared to habitual RFS runners (Miller et al., 2014; Kelly et al., 2018). However, it should be noted that other aspects of foot kinematics, such as toe flexion, that can influence plantar fascial strain.

The Achilles tendon and the triceps surae are the major contributors to energy storage and return during running (Alexander, 1994). Around 95% of the elastic strain energy stored in the Achilles tendon during the early stance is returned to propel the gait at late-stance (Peltonen et al., 2013). However, during the initial contact phase of RFS running, there is a rapid reduction in Achilles tendon force that is not present in FFS running (Komi, 1990). Thus, the Achilles tendon experiences higher loads in FFS running as they assist in dissipating much of the impact energy associated with eccentrically controlling the ankle dorsiflexion moment (Yong et al., 2020). Greater gastrocnemius activation in the eccentric phase, combined with high stretch velocity induces greater stiffness within the muscle-tendon unit in forefoot striking. This activation also results in an earlier and higher rate and magnitude (8%−24%) of Achilles tendon loading (Komi, 1990), which over time enhances its structural properties. Based on cadaveric studies, a 24% increase in Achilles tendon load with an FFS pattern would result in an additional 6 J energy returned by the tendon (Ker et al., 1987; Alexander, 1994). These results favor a FFS pattern when it comes to leveraging the Achilles tendon for energy return. In fact, habitual forefoot strike runners have greater Achilles tendon stiffness than habitual rearfoot strike runners (Wearing et al., 2019), and thus greater ability to store and release energy (Kyröläinen et al., 2003). This ability has been shown to reduce metabolic work and increase efficiency (Monte et al., 2020).

Sprinting is typically associated with a FFS pattern and sprinters have stiffer Achilles than distance runners (Arampatzis et al., 2007; Hatala et al., 2013). Running in minimal footwear has also promoted a FFS pattern. Runners habituated to this footwear exhibit greater stiffness and cross-sectional area of the Achilles tendon compared to those in conventional footwear (Histen et al., 2017). These studies collectively suggest that a habituated FFS pattern may invoke the necessary stimulus required for tendon adaptation and homeostasis, which leads to stronger calf muscles and Achilles tendons. Indeed, habitual FFS runners exhibit greater ankle plantarflexion strength than habitual RFS runners (Liebl et al., 2014). Future studies of habitual FFS runners are needed to determine whether the adaptations associated with an FFS pattern will result in fewer injuries to these structures.

### The Role of Intrinsic Foot Muscles in Locomotion

Intrinsic foot muscles are a bundle of small-volumetric muscles that originate and insert within the foot. Most of the intrinsic foot muscles lie beneath the dome of the foot skeleton and serve as the primary local stabilizers to the foot arch (Kelly et al., 2014). They act collectively to control the deformation of the foot arch (Soysa et al., 2012; Kelly et al., 2014; Miller et al., 2014; McKeon et al., 2015) and assist other leg muscles in actuating joint movements (McKeon et al., 2015; Zelik et al., 2015). The role of intrinsic foot muscles in human locomotion is mainly twofold. They maintain the integrity of the foot arch as a solid foundation for force production. Additionally, they allow certain compliance to arch deformation for energy recycling (Soysa et al., 2012). In the early stance, intrinsic foot muscles are mildly activated and tensioned (Kelly et al., 2015) to regulate segment movement and force transmission within the foot (Caravaggi et al., 2010; McKeon et al., 2015; Kirby, 2017). They become strongly engaged at push-off to consolidate the foot arch as an effective lever (Kelly et al., 2015), to which the calf muscle is anchored. This drives the body forward by producing large plantarflexion power (Lee and Piazza, 2009). Meanwhile, deformations of the foot arch and intrinsic foot muscles in the early stance absorb the impacts of landing and store elastic energy (Fukano and Fukubayashi, 2009; Kelly et al., 2019). This energy is later returned to propel the body when the foot arch recoils and intrinsic foot muscles contract (Soysa et al., 2012; Kelly et al., 2019). Evidence shows that the activation of intrinsic foot muscles can be enhanced to stiffen the foot arch in response to increased activity intensity (Kelly et al., 2012, 2014, 2015; Okamura et al., 2018). Weak or dysfunctional intrinsic foot muscles compromise gait performance and lead to injuries associated with foot deformities and tissue overload (Headlee et al., 2008; Huffer et al., 2017; Okamura et al., 2019; Taddei et al., 2020).

### How the Intrinsic Foot Muscles Function Differently in RFS and FFS

The most outstanding difference between RFS and FFS in kinematics is that the heel is off the ground in FFS at the initial contact of running (Morales-Orcajo et al., 2018). This position exposes the foot arch to a higher bending moment in FFS (Bruening et al., 2018; Kelly et al., 2018). The Achilles tendon force and ground reaction force act upward on the heel and forefoot respectively (Hashizume and Yanagiya, 2017; Rice and Patel, 2017). Concurrently, there is a compressive force from body mass applied over the top of the foot arch. Under this circumstance, FFS is estimated to increase arch compression and tensile stress within the plantar connective tissues (Chen et al., 2019a). However, many studies observe comparable foot arch deformation between RFS and FFS (McDonald et al., 2016; Wager and Challis, 2016). A possible explanation for the mismatch is that the intrinsic foot muscles are more activated in FFS to retain the foot arch height. Electromyography measurements show increased contract intensity and intensity duration of the intrinsic foot muscles in FFS compared to RFS (Kelly et al., 2018). This indicates that successful FFS has greater demands on foot muscle recruitment and foot strength. In this given, habitual RFSers are frequently suggested to complete a foot strength training program before transitioning to FFS (Mulligan and Cook, 2013; Davis et al., 2017; Taddei et al., 2020). Development of foot strength is considered a necessity to withstand burdens on the foot arch in FFS and protect the plantar soft tissues from painful pathologies, e.g., plantar fasciitis (Chen et al., 2019b). The greater activation of intrinsic foot muscles in FFS leads to greater arch stability and suggests that we were optimized for this strike pattern.

### Using Minimalist Shoes With FFS Strengthens the Foot Core Muscles

The past decade witnessed the revival of minimalist shoes in the running community. This was due to the thought that minimalist shoes reap the benefits of barefoot running, an allegedly more natural and healthier form of running (Franklin et al., 2018). Minimalist shoes are most characterized by the absence of motion-control and stability elements (Esculier et al., 2015). Due to this fact, minimalist shoes are expected to increase the involvement of foot structures in supplementing foot arch stability (Lieberman et al., 2010; Davis, 2014) and provoke a higher degree of intrinsic foot muscle activation in running (Franklin et al., 2018). Because landing on a cushionless heel is uncomfortable for habitual shod runners, minimalist shoes also promote a non-RFS running style (Lieberman et al., 2010; Squadrone et al., 2014), which adds to the requirements of strong foot musculature for injury-free usage (Bonacci et al., 2013). Research shows that runners having a healthy career with minimalist shoes exhibit stiffer foot arch and larger size of the intrinsic foot muscles (Holowka et al., 2018; Zhang et al., 2018). Therefore, many training protocols have been developed to help runners adopt minimalist shoes. The results demonstrate that running in minimalist shoes can stimulate the growth of intrinsic foot muscles, more specifically on the forefoot region (Chen et al., 2016), including the abductor hallucis and flexor digitorum brevis (Braunstein et al., 2005; Miller et al., 2014; Johnson et al., 2015; Campitelli et al., 2016). These muscles are particularly useful in stabilizing the longitudinal foot arch in a heel-rise position (Wong, 2007; Miller et al., 2014), correcting forefoot deformity (Xiang et al., 2018), and evening pressure distribution (McKeon et al., 2015) for running with minimalist shoes.

### The Interaction of Footwear and Footstrike

**Figure 2:**
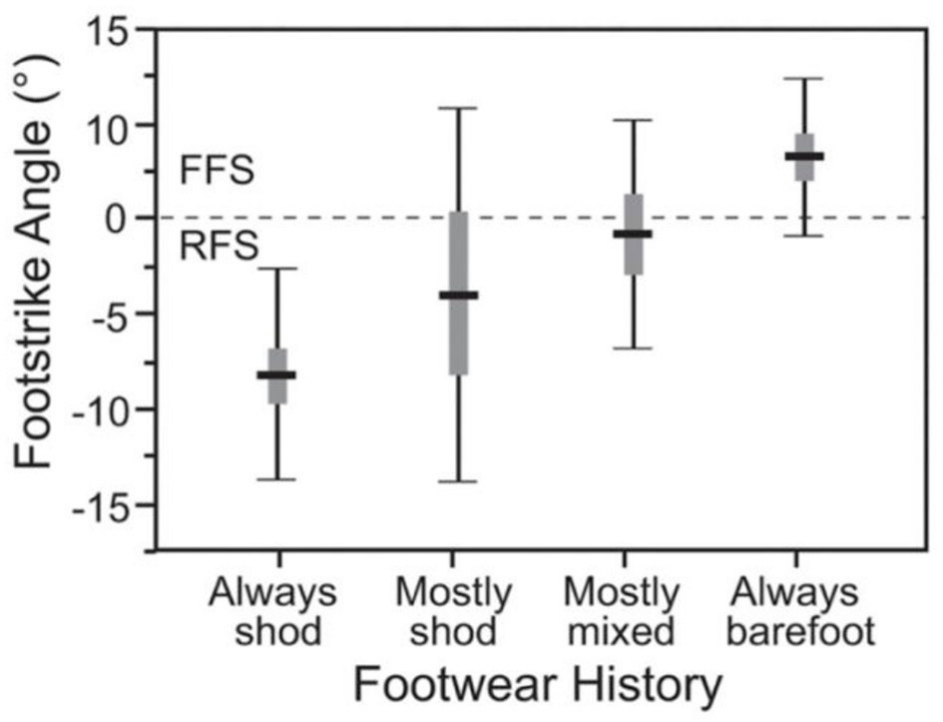
Relationship between footwear history and footstrike angle in Kenyan runners. Note that as footwear usage decreased, runners adopted a more forefoot strike (FFS) pattern. From Lieberman et al. (2015).

The work of Pai and Ledoux (2010) underscores the interaction of footwear and footstrike. These authors demonstrated that the more time a runner barefoot, the greater the tendency to run with a FFS pattern (Figure 2). A FFS pattern places greater demand on the calf muscles and Achilles tendon (Gruber et al., 2014), as well as the intrinsic foot muscles of the arch (Kelly et al., 2018). This demand requires greater activation of these muscles and therefore more muscular work. Placing a wedge of rubber under the heel allows a runner to land with a less demanding rearfoot strike pattern, and without pain that would likely occur with landing with a RFS barefoot. However, while it may take less calf work to land with a RFS pattern, this evolutionary mismatch results in harder landings with greater vertical impact forces leading to greater rates of loading (Pohl et al., 2009; Zadpoor and Nikooyan, 2011; Bonacci et al., 2013). These increased impacts and rates of loading have been associated with common running-related injuries (Pohl et al., 2009; Zadpoor and Nikooyan, 2011; Davis et al., 2016; Futrell et al., 2018; Johnson et al., 2020; Johnson and Davis, 2021).

The type of shoes also influences the interaction between footwear and footstrike. Running studies using conventional footwear have consistently shown that a FFS results in lower vertical load rates than a RFS. However, a FFS in this footwear results in greater anteroposterior and mediolateral impacts and load rates (Boyer et al., 2014; Rice et al., 2016) compared with a RFS in conventional footwear. Thus the resultant load rates of a RFS and FFS have been shown to be similar when running in traditional cushioned shoes. These shoes, with their elevated heels and flared outsoles, alter the natural FFS pattern by increasing plantarflexion and inversion at landing. However, when running with a FFS pattern in minimal shoes, all load rates are reduced resulting in the lowest resultant load rates compared with either a RFS or FFS in cushioned shoes (Rice et al., 2016). Therefore, there is a very important interaction between footstrike and footwear. Landing impacts in all directions will be lowest when running with a FFS pattern in minimal shoes. However, if a RFS pattern is preferred, there should be adequate cushioning under the heel. Additionally, these shoes should be replaced once the midsole loses its ability to cushion.

### Strike Pattern and Injury

While there are no prospective investigations of footstrike pattern and injury, retrospective and intervention studies have been conducted. A retrospective study of military recruits by Warr et al. (2015) reported no difference in injury patterns between RFS and non-RFS runners. However, in this study, runners with a midfoot strike (MFS) and FFS were combined into one group which likely confounds the results. Two papers (Ruder et al., 2019; Tenforde et al., 2020) have reported that that MFS vertical impact mechanics are similar to those of a RFS runner and are significantly different from a FFS runner. This suggests that MFS runners should either be grouped with RFS runners or considered separately. Therefore, results of the Warr et al. study need to be interpreted with caution. However, when looking at RFS and FFS separately, a retrospective study of the Harvard cross country team (Daoud et al., 2012) reported that RFS sustained twice the rate of repetitive stress injuries compared with FFS. In terms of interventions, shifting from a RFS to a FFS pattern has also been shown to be beneficial in terms of injuries. Diebal et al. transitioned 10 West Point cadets who had anterior compartment syndrome that was confirmed with intra-compartmental pressure testing (Diebal et al., 2011). Rather than undergo indicated fasciotomies, these cadets participated in a 6-week training program to adopt a FFS pattern. Following the program, compartment pressures were reduced to normal and pain, function and running performance were significantly improved. These improvements persisted at the 1-year follow-up as well. Most remarkable was all of the cadets were able to avoid the recommended surgery. In another intervention study, Roper et al. (2016) randomized 16 runners with patellofemoral pain into a control group and a FFS intervention group. Both groups gradually increased their treadmill runs from 15 to 30 min for 8 sessions over 2 weeks. However, the intervention group was provided real-time feedback to transition them to a FFS pattern. This feedback was faded during the last four sessions. Runners in the FFS group exhibited significant reductions in their pain. They also exhibited significant reductions in their patellofemoral contact stresses, an underpinning of patellofemoral pain (Liao et al., 2018). These improvements also persisted at the 1-month follow-up. This is an important finding given that patellofemoral pain is one of the most common injuries runners sustain. These latter three studies collectively suggest that running with a FFS pattern may be associated with lower rates of common running-related injuries.

### Transitioning

As a result of becoming reliant on the cushioning of modern footwear that has led to a habitual RFS pattern, a gradual transition program is needed to safely shift to a FFS pattern. As a FFS is best accomplished in minimal footwear, a slow transition becomes even more important. As previously noted, both the FFS pattern and minimal footwear, require greater activation of the calf and arch musculature. This will increase the demand on these tissues and will place them at risk if the transition is not done slowly. A gradual transition can simply be a function of slowly increasing mileage in the FFS condition (Zhang et al., 2021), allowing the tissues to adapt accordingly to the new load. This can be accomplished with a simple walk-run program where running gradually replaces walking (McCarthy et al., 2013; Chen et al., 2016). However, the addition of a strengthening program for the calf and feet helps to increase the capacity of the musculoskeletal system (Mulligan and Cook, 2013; Futrell et al., 2020; Taddei et al., 2020), thereby further reducing the chance of an overuse injury.

### Final Thoughts

We have presented evidence suggesting that running with a RFS pattern is an evolutionary mismatch with the way we were adapted to run. We have supported this thesis by providing evidence of foot and ankle structure and function that is optimized for FFS. Transitioning to a FFS pattern shifts some of the mechanical work done from the vulnerable knee to the foot and ankle for which we propose it is adapted. However, if these structures have become deconditioned from supportive footwear and a habitual RFS pattern, time is needed to develop the capacity of these structures. Prospective studies are still needed to compare the long-term risk, beyond the transition period, of musculoskeletal injuries between RFS and FFS runners. The holy grail may be with our children by starting them in shoes that do not influence running mechanics (no support, cushioning, flares, etc.). This will allow the development of the running pattern of our ancient ancestors and hopefully reduce the risk of future lower extremity musculoskeletal injuries.

## Data Availability Statement

The original contributions presented in the study are included in the article/supplementary material, further inquiries can be directed to the corresponding author.

## Author Contributions

ID: primary responsibility for manuscript, wrote all sections except for those listed below, reviewed, and edited entire manuscript. TC: wrote sections on intrinsic foot muscles, reviewed, and edited entire manuscript. SW: wrote sections on tissue characteristics, reviewed, and edited entire manuscript. All authors contributed to the article and approved the submitted version.

## Conflict of Interest

The authors declare that the research was conducted in the absence of any commercial or financial relationships that could be construed as a potential conflict of interest.

## Publisher's Note

All claims expressed in this article are solely those of the authors and do not necessarily represent those of their affiliated organizations, or those of the publisher, the editors and the reviewers. Any product that may be evaluated in this article, or claim that may be made by its manufacturer, is not guaranteed or endorsed by the publisher.

## Footnotes

^1^Murphy S. “Heel striking – is it really the enemy of good running form?” The Guardian. Available online at: https://www.theguardian.com/lifeandstyle/the-running-blog/2014/oct/09/is-heel-striking-the-enemy-of-good-running-form (accessed September 20, 2021).

^2^Dunne J. “Is forefoot running better for your knees? – Running technique.” Kinetic Revolution – Run Strong. Available online at: https://www.kinetic-revolution.com/is-forefoot-running-better-for-your-knees/ (accessed September 20, 2021).
